# Late stage inhibition of hematogenous melanoma metastasis by cystatin C over-expression

**DOI:** 10.1186/1475-2867-5-14

**Published:** 2005-05-17

**Authors:** Heather Ervin, James L Cox

**Affiliations:** 1 Department of Biochemistry, Kirksville College of Osteopathic Medicine, 800 West Jefferson, Kirksville, Missouri 63501-1497 USA

## Abstract

**Background:**

Tumor metastasis is a frequent cause of treatment failure for cancer patients. A key feature of metastatic cancer cells is their invasive ability. Cysteine proteases contribute to invasive properties of many cancer cell types. To analyze the contribution of cysteine proteases to metastasis we have over-expressed in B16 melanoma cells the natural cysteine protease inhibitor, cystatin C. We measured *in vitro *invasion of cystatin over-expression clones with Boyden chamber type assays. Tail-vein injections of cells were used to compare lung tumor colonization. Subcutaneous tumor growth and tumor cell metastasis from primary tumors were also analyzed. Apoptosis of tumor cells was measured in lung tissues following melanoma cell injection.

**Results:**

Results show the *in vitro *invasion of cystatin C over-expressing cells was dramatically inhibited. Lung tumor colonization was also reduced. Increased tumor cell apoptosis was found to be an important factor and may be related to the reduced tumor burden noted in this system of melanoma metastasis.

**Conclusion:**

Cysteine proteases therefore, may be a target for future anti-metastatic therapies.

## Background

Tumor metastasis is a major reason for treatment failure in cancer patients. Invasion of host tissues is a hallmark feature of metastasis and requires alterations in tumor cell adhesion, cell migration, and proteolytic degradation of tissue matrices [1,2]. A critical initial step of metastasis is that tumor cells enter blood vessels or the lymphatic system by invasion of host basement membranes. After being dispersed through vascular spaces, metastatic tumor cells lodge in distant capillaries. Late stages of metastasis involve further steps including invasion from vascular spaces into foreign tissues and growth of secondary metastases in permissive environments (seed and soil hypothesis) [2]. Growth of metastases in tissues also requires invasion of host endothelial cells into tumor masses to support further the metabolic needs of tumor growth [3].

Members of the papain cysteine protease family of enzymes (primarily cathepsins B, H, and L) are elevated in cancer cells and contribute to invasion of many tumor types [4,5]. This high cysteine protease activity influences the activity of other proteolytic enzymes during acquisition of an invasive phenotype [6]. Cysteine proteases help create an invasive phenotype at one or more steps in tumor progression to metastasis [7]. Details are lacking in the exact roles of cysteine proteases in tumor progression, but it is clear that highly metastatic variants of various tumor types express an excess of protease activity of the papain cysteine protease type [8]. Because elevated cysteine protease activity correlates well with highly invasive cancers, inhibition of this activity may be a target for anti-metastatic therapies.

Cystatins are potent inhibitors of cysteine proteases that are found in all tissues and most biological fluids. In general, a decrease in cysteine protease inhibitor levels is also found in metastatic tumors, contributing to higher cysteine protease levels [9]. Cystatin C is a type II cysteine protease inhibitor that is normally secreted from cells [10]. Previously we have investigated the role of the natural cysteine protease inhibitor, cystatin C, and found inhibition of lung metastasis [11]. To help establish mechanism of cystatin C action in metastasis we have fused cystatin C to the N-terminus of green fluorescent protein. In this study we show over-expression of cystatin C in melanoma cells is associated with reduced metastasis and increased apoptosis in lung tissues.

## Results

### Expression of cystatin C-GFP fusion

**Figure 1:**
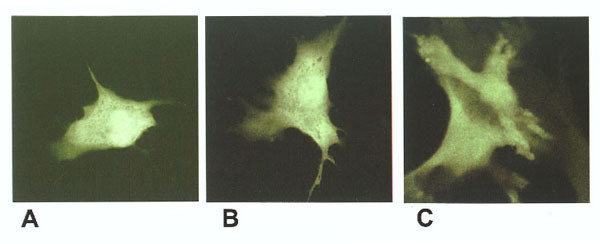
*GFP fluorescence*. B16 melanoma cells transfected with cystatin C-GFP fusion construct A, B) transiently transfected B16F10C) stably transfected B16F10 cells.

**Figure 2:**
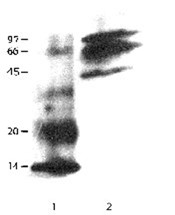
*Western blot analysis of cystatin C-GFP fusion*. Lane 1, human cystatin C, 1 ug; lane 2, c23 fusion clone (40 ug/lane total cell extract). Probed with anti-human cystatin C antibody. Fusion is 14 kD cystatin C plus 30 kD GFP protein ~44 kD. Multimers form with denatured cystatin C.

The expression of cystatin C-GFP fusion was observed in B16F10 melanoma cells following transient transfection of plasmid construct DNA. Confocal microscopy was used in an attempt to localize cystatin C-GFP protein expression in cells. A cytoplasmic expression was noted for GFP alone and cystatin C-GFP fusion when transiently expressed in B16 melanoma cells (Figure 1). This pattern of expression was not anticipated since cystatin C is primarily a secreted protein that should have produced a vesicular localization of fluorescence within the cell. Figure 2 shows expression of the cystatin C-GFP fusion protein. Cystatin C fused to GFP should be about 44 kD (30 kD GFP plus 14 kD cystatin) and has also formed multimers under denaturing conditions. Recently Paraoan et al. have described cytoplasmic distribution of human cystatin C mutated within the signal peptide [16]. Similarly, sequence analysis of RT-PCR product revealed a signal peptide amino acid difference (-4 Ala→Gly) between Balb c (inserted cDNA) and B16 melanoma cystatin C messenger RNA (endogenous) (data not shown). We recloned the cystatin C-GFP fusion and re-transfected the DNA construct and obtained identical results. We believe cystatin C over-expression effects are primarily due to intracellular expression in our B16 melanoma cell model.

### Cysteine protease activity

The total cellular cysteine protease activity was measured with a sensitive fluorometric assay. This assay indirectly measures protease inhibitor levels in the cell as cystatins are often found at low levels. Table 1 shows decreased cysteine protease activity in cystatin C over-expression clone c23. Most of the cellular cysteine protease activity is localized to lysosomes that would be unaffected by altered cystatin levels.

**Table 1:** Cellular cysteine protease activity in vitro

**Clone**	Activity*	**Relative activity %**
**G1**	20 +/- 0.8	100
**c23**	15.6 +/- 0.7	78

*Arbitrary fluorescence units. Experiment carried out 3 times. Students t-test, p < .05.

### *In vitro *invasion of B16 melanoma is inhibited by cystatin C over-expression

**Figure 3:**
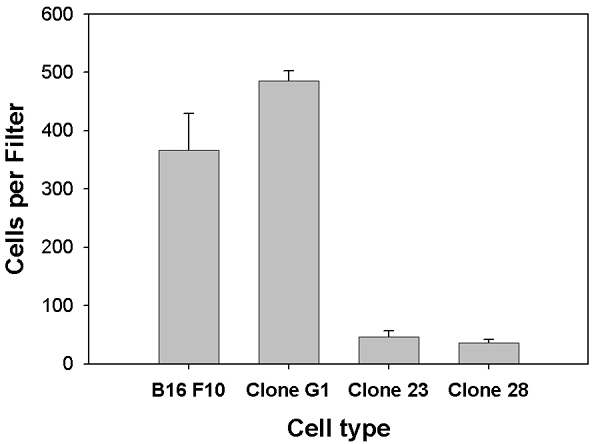
*Invasion assay*. Boyden chamber type assays comparing cell line B16 F10, clone G1 (GFP-expressing), c23 and c28 (cystatin C-GFP fusion) invasion through Matrigel. Experiment was run in triplicate with three filters for each clone per experiment. Data is expressed in cells / filter ± s.d. Student's t test P < .001.

One important characteristic of metastasis is the invasive ability of tumor cells. We used an *in vitro *Boyden chamber assay to quantify the invasive potential of our clonal lines. [17]. Over-expression of cystatin C-GFP (c23, c28) decreased the invasion of B16F10 melanoma cells through Matrigel coated filters by about 90% relative to control G1 cells (GFP transfected) (Fig 3). Cystatin-GFP fusion clones behave similarly to cystatin over-expressing clones without the GFP fusion. Growth rate is the same as control; adhesion is the same as control; and cell migration is reduced in comparison to control ([12] and personal observations)

### Cystatin C over-expression reduces lung colonization and increases survival in mice

**Figure 4:**
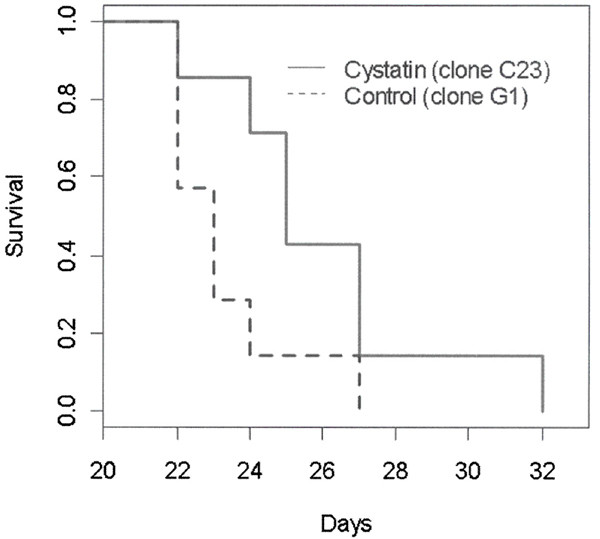
*Survival curve*. Comparison of survival of mice injected via tail-vein with either clone G1 or c23 cells (7 mice per group).

We examined the effect of cystatin C over-expression on lung tumor growth and overall survival of mice injected via tail vein. Tail vein injections are measures of experimental or late stage metastasis only because the initial invasion and intravasation are bypassed. We hypothesized that the large decrease measured for *in vitro *invasion might lead to similar decreases in lung colonization in mice. Although we measured a decrease in lung tumor burden following tail vein injection in the cystatin C over-expressing clone, this clone still resulted in tumor colonization (Table 2). We next examined the effect of cystatin C over-expression on survival of mice injected with B16F10 melanoma cells and found about a 10% increase in median survival time (Figure 4). Our conclusion from this data is that over-expression of cystatin C, while decreasing tumor burden, causes only modest increase in the survival of animals following tail vein injection of melanoma cells. We show that a large decrease in melanoma invasive ability with cystatin over-expression does not by itself prevent subsequent growth of metastases.

**Table 2:** Lung tumor colonization by B16 F10 transfected cell lines

	**Tumors / Lung**	
Clone*	**Average.**	**Range**

**G1**	25 ± 12	12–45
**C23**	14 ± 5	5–23

* n = 6 mice for each clone

### Subcutaneous growth of B16 melanoma with cystatin C over-expression is similar to control

**Figure 5:**
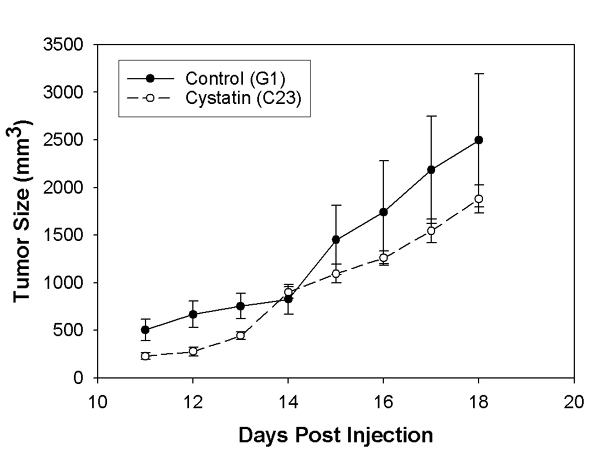
*Subcutaneous tumor growth*. Comparison of tumor volume of clone G1(control) or c23 (cystatin over-expressor). (4 mice per group) Paired Wilcoxon test P < 0.01.

We compared the subcutaneous growth of B16 melanoma control and cystatin C over-expressing clones in syngeneic C57 BL6 mice. Following subcutaneous, scapular injection of B16 melanoma tumor cells, tumors become measurable by calipers after about ten days. Figure 5 shows the relative growth of subcutaneous tumors in mice. A slight decrease in subcutaneous tumor growth was noted for a cystatin C over-expression clone. In a separate experiment we compared the relative lung metastasis of melanoma cells originating from subcutaneous tumors. These secondary lung metastases were quantitated at 14 days following subcutaneous injection of either clone G1 or clone c23. After sacrifice of the mice, tumor burden was estimated by S100 staining of frozen lung tissue cross-sections. We found a significant reduction in lung metastasis from primary subcutaneous tumors upon cystatin C over-expression (Table 3).

**Table 3:** Melanoma metastasis to lung from subcutaneous primary tumors

**Clone**	Average area ± s.e.m. (%_total)*
G1	26.0 ± 4.9
C23	4.3 ± 1.4

* Imaged S100 antibody positive staining for 4 tumors with 3 sections per tumor and three fields per section. (P = .005 for Student's t test).

### Apoptosis of micrometastases is increased with cystatin C over-expression

**Figure 6:**
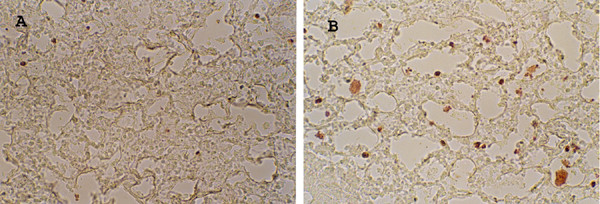
*Apoptosis*. Representative lung tissue sections stained for apoptosis with TUNEL reagents. **A**. Control (G1) cell injected mouse. **B**. c23 (cystatin C over-expressor) cell injected mouse.

We examined the distribution of both GFP labeled cells and cystatin C-GFP labeled cells in lung tissue at 24 hours following tail vein injection. The cystatin C-GFP fusion product that we introduced into B16 F10 melanoma allowed us to follow the distribution of metastatic cells in animals. The melanoma cell density in the lung and melanoma distributions were similar for both GFP labeled and cystatin C-GFP clones (data not shown). This observation suggests delivery of tumor cells for each clone to lung capillaries is equivalent and cannot account for differences in subsequent lung tumor burden. We instead focused on tumor growth in late stage metastasis. An examination of melanoma cell apoptosis at early times after lung tissue seeding of melanoma cells provides insight into tumor outgrowth at later stages. One week after melanoma cell tail-vein injection, we excised lung tissue and examined the levels of apoptotic cells. We used the Dead-End TUNEL Assay to quantitate apoptotic cells in frozen tissue sections. Figure 6 shows typical tissue sections stained for apoptotic cells at one week post-injection. Quantitation of apoptosis showed an average two-fold increase in apoptotic melanoma cells within lung tissue of animals injected with cystatin C over-expressing cells (Table 4). Thus, increased apoptosis of lung metastasized B16 melanoma cells correlates with decreased tumor burden upon cystatin C over-expression.

**Table 4:** Apoptosis in lung tissue sections

		**Apoptotic cells/field**		
	**Mouse**	**Clone G1**	**Clone c23**	**G1/C23 ratio**

**Expt 1**	**1**	36 ± 3	78 ± 9	2.2
	**2**	8.6 ± 1.5	44 ± 7	5.1
	**3**	11.6 ± 1.3	25 ± 2.0	2.1
	**4**	13.7 ± 2.0	26 ± 4.3	1.9

				**2.8 average**

	**Mouse**	**Clone G1**	**Clone c23**	**G1/C23 ratio**

**Expt 2**	**1**	10.5 ± 1.6	35.8 ± 2.5	3.4
	**2**	8.6 ± 0.8	20 ± 4.3	2.3
	**3**	6.5 ± 0.8	12.2 ± 1.6	1.9

				**2.5 average**

## Discussion

Blockade of the metastatic spread of cancer could provide dramatic clinical improvements for the treatment of cancer patients. The therapeutic blockade of metastasis is a formidable challenge due to many factors including redundant invasion mechanisms of metastatic cells, acquisition of chemotherapeutic resistance, and evasion of host immune responses. In spite of these problems, inhibition of metastatic cell invasion could be a possible adjunctive treatment for metastatic disease. We have previously found cystatin C over-expression inhibits both melanoma cell migration and metastasis [12]. In this work, expression of cystatin C fused to GFP also showed a dramatic inhibition of *in vitro *invasion of B16 melanoma cells. The number of lung metastases following tail-vein injection were decreased, however growth of subcutaneous melanoma tumors over-expressing cystatin C was only slightly inhibited. The expression of cystatin C fused to GFP we obtained exhibited primarily cytosolic localization. Another group has reported cytosolic localization of GFP-labeled cystatin C due to a signal peptide mutation [16]. We have noted an amino acid change near the signal peptide cleavage site and suggest abnormal cellular distribution may result. The point is, our results on melanoma metastasis may only pertain to intracellular over-expression of cystatin C.

We have demonstrated cystatin over-expression in melanoma cells appears to result in an increase in apoptosis in lung micrometastases *in vivo*. The growth of secondary metastases is considered to be rate limiting in the metastatic cascade of tumor spread [18,19]. Over-expression of the metalloprotease inhibitor TIMP-1 does not block B16 melanoma cell extravasation into lung tissue, but does alter subsequent tumor growth within lung tissue [20]. Our work supports the idea that inhibition of cell invasion alone is not sufficient to prevent lung tissue colonization by metastatic cells [21]. In this work we demonstrate that apoptosis is increased in lung micrometastases when melanoma cell invasion is inhibited by cystatin C over-expression. Increased tumor cell apoptosis may be the result of alterations in the tumor cell microenvironment *in vivo*, for example, alterations in tumor cell adhesion or local release of growth factors required for tumor cell survival. Inhibition of angiogenesis is probably not involved because after at one-week analysis, virtually all micrometastases were only a few cells in diameter. Generally, angiogenesis begins only as tumor size approaches 1 mm [22].

Additional effects cystatin on melanoma cell metastasis might have been expected if our melanoma cystatin over-expressing clones had secreted cystatin C. Multiple roles extracellular cathepsin B have been suggested, including matrix degradation [23]. A synthetic inhibitor of cysteine proteases has been found to decrease metastasis in a colon cancer model, however the point of metastatic block was not defined [24]. Recently, Konduri et al. [25] have shown over-expression of cystatin C in glioblastoma cells blocks tumor cell invasion and tumor growth *in vivo*, however in their system cystatin C was secreted.

## Conclusion

Metastatic melanoma is a rapidly spreading and growing cancer type that thwarts virtually all attempts at growth arrest. Blockade of metastasis would improve current treatments of melanoma that are primarily targeted at tumor cell proliferation. We have found that cystatin C over-expression dramatically reduces melanoma cell invasion. Late stage metastasis is influenced for cystatin C over-expressing cells by an apparent increased apoptosis in the target tissue (in this case, lung). Our results indicate that inhibition of cell invasion alone is insufficient to dramatically improve the survival of mice at late stage melanoma metastasis. More favorable results may occur at earlier steps of metastasis intervention with anti-invasive agents or with combined therapies that target tumor growth and /or angiogenesis.

## Methods

### Cell culture

The B16-F10 melanoma cell line was kindly provided by Dr. I. Fidler (MD Anderson Cancer Center, Houston, TX). Cells were grown in culture on plastic dishes as monolayers in RPMI-1640 media containing 10% fetal bovine serum (v/v) and supplemented with antibiotics (100 units penicillin, 0.1 mg streptomycin, 0.25 μg amphotericin B per milliliter) (Sigma). Cells were cultured in a 5% CO_2 _humidified atmosphere at 37°C until near confluence.

### DNA constructs and cell transfections

Murine cystatin C cDNA was described in a previous paper [12]. Fusion of cystatin C cDNA to the N-terminus of green fluorescent protein (GFP) (Invitrogen) was carried out with a fusion fragment comprised of two annealed oligos (5'-CTGCAAAAATGCCA-3', 5'-CGTGGCATTTTTGCAG3') (Integrated DNA Technologies, Corallville, Iowa). Ligated DNA was transformed into DH5a *E. coli *cells by electroporation with a Biorad Gene Pulser following the manufacturer's instructions. Fusion clones were confirmed by restriction analysis and lysis-purified DNA was used for transfection of B16 melanoma cells. Transfection of plasmid DNA into B16 F10 melanoma cells was carried out by the calcium phosphate method [13]. Transiently transfected cells were imaged by confocal microscopy one or two days after transfection. Stable transformants were selected in media containing G418 (geneticin)(Sigma) at 1 mg/ml. Three clonal lines were chosen for study after three weeks selection: a GFP-control clone (designated G1) and two cystatin over-expressing clones (designated c23 and c28).

### Western blot analysis

B16 melanoma F10 clone c23 was grown to near confluence and lysed with buffer (0.1% Triton X-100 in PBS with 1% protease inhibitors (Sigma)) through three freeze thaw cycles and centrifuged at 10 krpm for 5 minutes to pellet cell debris. Cell extracts (40 ug) were run on 10% SDS PAGE gels and electro-blotted to PVDF membranes for antibody detection. Human cystatin C (1 ug) (Calbiochem) was run as a control. Rabbit anti-human cystatin C (Upstate Biotechnology) was used at 1:1000 dilution. Secondary antibody, goat anti-rabbit HRP (Upstate Biotechnology) was used at 1:1000 and detection was by ECL luminescence system. (Amersham). Protein was determined by the Bradford assay.

### Assay of cysteine protease activity

We used a fluorometric assay to measure total cellular cysteine protease activity [14]. This assay indirectly measures cysteine protease inhibitor levels that are difficult to measure directly due to low levels. Either G1 (GFP) or c23 (cystatin C-GFP fusion) cells were seeded at 5 × 10^4 ^cells per well in a 24 well cell culture plate. After 16 hours growth the cells were incubated with PAB buffer (Hank's buffered saline plus 0.6 mM CaCl_2_, 0.6 mM MgCl_2_, 2 mM cysteine, and 25 mM PIPES pH7.0) for 30 minutes at 37°C. The buffer was then replaced with 0.2 ml of the same buffer containing 0.1% Triton X-100 and 100 uM Z-Phe-Arg AMC (Enzyme Systems Products, Livermore, CA). After 20 minutes further incubation at 37°C, fluorescence was read in a BioTek flx800 fluorescent plate reader at 360 excitation/460 emission and the results were expressed in relative fluorescence units. The assay was found to be linear for 30 minutes under the conditions used.

### Cell invasion

Sub-confluent B16 F10 cells or permanently transfected B16 melanoma clones were detached with trypsin/EDTA solution (0.25%, 5 minutes) (Sigma). Following inactivation of trypsin by addition of an equal volume of serum-containing media, the cells were counted with a hemocytometer. Cells (2 × 10^4^) were placed into top wells of Boyden chambers (BD Biosciences), the filters (8 um pore size, Osmonics, Inc.) pre-coated with Matrigel (65 ug/filter) [12]. Cell invasion was allowed to occur for 24 hours, after which time cells were fixed and stained [methanol, 10 minutes; 0.1% Triton X-100, 2 minutes; Harris hematoxylin (Sigma), 10 minutes]. After 24 hours, cells on the bottom of the filter were counted with an inverted Olympus IMT-2 microscope at 400× magnification. Cell numbers for 18 fields were summed for each filter.

### Survival curve

An experiment was conducted where mice (seven mice per group) were tail vein injected (1 × 10^5 ^cells per mouse) with either control GFP (G1) or cystatin C-GFP fusion clone (c23). The mice were followed until time of death or were moribund in which case mice were sacrificed.

### Lung metastasis-assay

Clones G1 and c23 were grown to near confluence and detached with trypsin solution. Trypsin was neutralized with an equal volume of cell culture media and washed twice with phosphate buffered saline (PBS). Cells were re-suspended in PBS and 1 × 10^5 ^were injected into tail veins of C57BL6 female mice (seven per group). After 30 days mice were sacrificed and lung tissues were excised. Lung surface tumors were counted under a low-power dissecting microscope.

### Subcutaneous tumor growth

Cells of clones to be tested (G1 and c23) were grown to near confluence, detached with brief trypsin treatment, and washed twice with PBS. After re-suspension in PBS, the cells (3 × 10^5^) were injected subcutaneously into scapular regions of C57BL6 mice (six mice per group). When tumors began to appear (after about ten days), tumor diameter was measured on successive days in two orthogonal directions with calipers. Tumor volumes were calculated by the formula V = (a^2 ^× b)/2, were a = width and b = length, in millimeters [15].

### Lung metastasis assay from subcutaneous tumors

C57BL6 mice (6 mice per group) were injected subcutaneously with either clone G1 or clone c23 cells at 2 × 10^5 ^cells per mouse. After 3 weeks mice were sacrificed and lungs were removed and frozen in OCT media for sectioning. Frozen tissue sections (10 uM) from each lung tissue sample were stained with S100 antibody (DAKO, 1:1000 dilution) and immunologically detected with a secondary horseradish peroxidase antibody (Sigma, 1:1000). Microscopic images (100×) of stained tissue sections were collected from 4 mice in each group. Quantitation of stained area per section was carried out with Image J (NIH software).

### Apoptosis of lung tumor cells

Mice (C57BL6, 3–4 animals per group) were injected with B16 F10 clones G1 (GFP), or c23 (cystatin C-GFP) at 1 × 10^5 ^cells via tail vein. After one week mice were sacrificed and lung tissue was removed. Lung tissues were stored in OCT embedding compound (Miles Inc.) overnight at 4°C and then frozen at -80°C. Frozen tissue sections (10 um) were prepared and stained for TUNEL reactivity with the Dead-End TUNEL kit (Promega) directly on glass coverslips. Manufacturers instructions were followed with side-by-side staining of G1 and c23. Stained cells were summed for at least five 200× microscopic fields per tissue section and six independent sections per animal. The average number of apoptotic cells per high powered field were recorded ± s.d.

## List of abbreviations

GFP, Green fluorescent protein; AMC, 7-amino-4-methylcoumarin; PBS, Phosphate buffered saline; ECL, enhanced chemical luminescence.

## Competing interests

The author(s) declare that they have no competing interests.

## Authors' contributions

HE carried out the apoptosis assays, western blot analysis, and cell maintenance. JC conducted the remaining procedures and drafted the manuscript.
